# Cicadidae Periostracum Attenuates Atopic Dermatitis Symptoms and Pathology via the Regulation of NLRP3 Inflammasome Activation

**DOI:** 10.1155/2021/8878153

**Published:** 2021-01-13

**Authors:** Gunhyuk Park, Byeong Cheol Moon, Seung Mok Ryu, Wook Jin Kim, Hye-Sun Lim

**Affiliations:** Herbal Medicine Resources Research Center, Korea Institute of Oriental Medicine, 111 Geonjae-ro, Naju-si 58245, Republic of Korea

## Abstract

Atopic dermatitis (AD) is a multifactorial inflammatory skin disease of complex etiology. Despite its increasing prevalence, treatment for AD is still limited. Crude drugs, including herbal extracts or natural resources, are being used to treat AD symptoms, with minimum side effects. Cicadidae Periostracum (CP), derived from the slough of insects belonging to the family Cicadidae, is a commonly used crude drug in traditional Asian medicine to treat/control epilepsy, shock, and edema. However, the effect of CP on AD-like skin lesions is unknown. In this study, we examined the effect of a CP water extract on AD disease development *in vivo*, using a house dust mite-induced AD mouse model, and *in vitro*, using HaCaT keratinocytes and a 3D human skin equivalent system. Importantly, CP administration alleviated house dust mite-induced AD-like symptoms, suggested by the quantified dermatitis scores, animal scratching behaviors, skin moisture retention capacity, and skin lesion and ear thickness. Furthermore, histopathological analysis demonstrated that CP decreased intralesional mast cell infiltration. In addition, CP treatments decreased the systemic levels of immunoglobulin E, histamine, and thymic stromal lymphopoietin (TSLP) and the local mRNA expression of TSLP and several Th1/Th2 cytokines. Our data suggest that these effects were mediated by the inhibition of nucleotide-binding oligomerization domain-like receptor protein 3 (NLRP3) inflammasome activation. *In vivo* and *in vitro* CP treatments resulted in the downregulation of inflammasome components, such as ASC and cleaved caspase-1, as well as related mediators such as IL-1*β* and reactive oxygen species. Collectively, our results suggest that CP is a potential therapeutic agent for AD, controlling inflammatory responses through the suppression of NLRP3 inflammasome activation.

## 1. Introduction

Atopic dermatitis (AD) is a multifactorial inflammatory skin disease of complex etiology, resulting from the interaction of genetic, environmental, and psychological factors [1]. AD is characterized by the appearance of eczematous lesions, with pruritus and dry skin, associated with the disruption of epidermal barriers, abnormal immune responses, and immunoglobulin (Ig) E secretion [1]. AD onset is attributed to hypersensitive immune cells, including keratinocytes, monocytes, and dendritic cells, which overreact to environmental agents, such as house dust mite allergens, food, and bacteria, leading to overwhelming inflammatory responses [1, 2]. The pathogenesis of AD involves the disruption of T helper (Th) 1/Th2 cytokine homeostasis towards Th2 skewed immune responses [2, 3]. When Th2 cells are activated, cytokines such as thymic stromal lymphopoietin (TSLP), interleukin- (IL-) 4, IL-5, IL-10, and IL-13 are secreted, enhancing humoral immune responses and inhibiting the function of Th1 cells [3].

House dust mites are well-known allergens. *Dermatophagoides pteronyssinus* and *Dermatophagoides farinae* are the most common house dust mite species in temperate climates and have been associated with the pathogenesis of AD via IgE recognition [4, 5]. The innate immune system is activated via evolutionarily conserved pathogen recognition receptors, such as Toll-like receptors and nucleotide-binding oligomerization domain-like receptors (NLRs) [6]. NLR family pyrin domain-containing protein 3 (P3) is a widely recognized NLR, is part of an important inflammasome, and is also composed of apoptosis-associated speck-like protein containing a caspase recruitment domain (ASC) and of pro-caspase-1 [7, 8]. Inflammasome assembly is triggered by NLRP3 activation and leads to caspase-1 activation, which in turn promotes the maturation and release of cytokines such as IL-1*β* and IL-18 and the initiation/amplification/regulation of multiple immune responses [9]. In fact, the NLRP3 inflammasome is a recognized key regulator of contact hypersensitivity [10]. A recent study linked the NLRP3 inflammasome to allergic diseases, revealing an association between two NLRP3 single nucleotide polymorphisms and increased susceptibility to food-induced anaphylaxis and aspirin-induced asthma development [11, 12]. Therefore, NLRP3 inflammasome regulation may represent a novel therapeutic approach for the treatment of allergic diseases, including AD.

Cicadidae Periostracum (CP) is a crude drug composed of nymphal exuviae of *Cryptotympana atrata* (Fabricius, 1775) also known as “Cicadas” or “Sun-Tae,” originally described in an ancient Korean medical book, the *Dongui Bogam*, within the Chung-bu category [13, 14]. CP has been used to treat epilepsy, shock, smallpox, sedation, edema, and night terror symptoms in Korean traditional medicine [14]. Moreover, in traditional Chinese medicine, CP is known as “Chantui” and has long been used to treat soreness of the throat, hoarseness, itching, and spasms, among other symptoms [15, 16]. Recently, we reported the CP effectiveness in an MPTP-induced Parkinson's disease mouse model, via the regulation of the nurr1 gene [17]. Additionally, previous studies reported that the Periostracum cicada inhibits oxidative stress and inflammation caused by UVB irradiation in HaCaT keratinocytes [18]. However, scientific evidence for the use of CP in skin diseases, including AD, is still insufficient.

In this study, we investigated the potential of CP as a treatment for AD-like symptoms, using a house dust mite-induced AD mouse model. Furthermore, we tried to disclose the potential CP mechanisms of action, both *in vitro* and *in vivo*. Special attention was paid to the potential effects exerted on NLRP3 inflammasome activation.

## 2. Materials and Methods

### 2.1. Preparation of the CP Extract

A standardized CP extract was prepared according to previously published methods and supplementary materials [17].

### 2.2. Experimental Animals and Ethics Statement

Male, eight-week-old NC/Nga mice were purchased from Central Laboratory Animal Inc. (Seoul, Korea) and maintained under temperature- and light-controlled conditions (22 ± 2°C, 12 h light-dark cycle) with food and water provided *ad libitum*. All experimental procedures were approved by the Korea Institute of Oriental Medicine Animal Care and Use Committee (approval number: #19-039) and performed in accordance with the relevant guidelines and regulations.

### 2.3. Induction of Experimental AD and Experimental Layout

**Figure 1:**
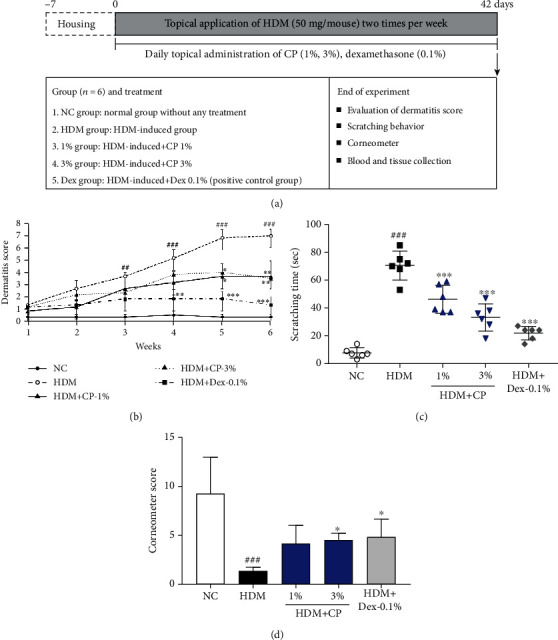
CP treatments alleviate AD-like symptoms in a house dust mite-induced AD mouse model. (a) Schematic diagram of the experimental protocol. (b) Dermatitis scores were evaluated weekly. (c) Scratching frequency and (d) skin moisture retention were evaluated on day 42 postsensitization. Dexamethasone (Dex) was used as a positive control. All data are represented per group, as means ± SEM (*n* = 6 mice per group). Statistical differences were evaluated using the one-way ANOVA test, with Dunnett's post hoc analysis, and are represented as follows: ^##^*P* < 0.01 and ^###^*P* < 0.001 compared with the negative control group (NC); ^∗^*P* < 0.05, ^∗∗^*P* < 0.01, and ^∗∗∗^*P* < 0.001 compared with the untreated control group (HDM).

To induce barrier integrity disruption, 200 *μ*L of a 4% sodium dodecyl sulfate solution was applied to the previously shaved dorsal skin samples and the ventral and dorsal ear surfaces of NC/Nga mice. Experimental AD-like skin lesions were then induced by the topical application of 50 mg of house dust mite ointment (Biostir-AD; contains *Dermatophagoides farina* extract; Biostir Inc., Osaka, Japan) twice per week, for 6 weeks. The experimental layout is summarized in Figure 1(a). Mice were divided into five groups (*n* = 6 per group): (1) negative control (NC; vehicle-treated mice; distilled water), (2) untreated control (house dust mite+distilled water), (3) CP 1% treatment (house dust mite ointment+CP 1%), (4) CP 3% treatment (house dust mite ointment+CP 3%), and (5) Dex-0.1% treatment (house dust mite+dexamethasone 0.1%). CP and dexamethasone (Sigma-Aldrich, St. Louis, MO, USA) were dissolved in distilled water and applied every day for 6 weeks. Dexamethasone was used as a (treatment) positive control.

### 2.4. Experimental Endpoints and Euthanasia

Blood was collected by the aorta ventralis and left to clot, and serum was separated by centrifugation at 5000 × *g* for 10 min at 4°C and stored at -80°C for posterior analysis.

### 2.5. Evaluation of Dermatitis Scores

The relative severity of dermatitis was assessed macroscopically according to the Eczema Area and Severity Index (EASI) scoring system: 0, no symptoms; 1, mild symptoms; 2, moderate symptoms; and 3, severe symptoms. The dermatitis scores were defined as the sum of erythema/hemorrhage, edema, excoriation/erosion, and scaling/dryness scores [19]. Mice were photographed once each week, for 6 weeks, with a digital camera (Canon EOS 5D Mark IV, Canon Inc., Tokyo, Japan). Clinical evaluation was based on these pictures.

### 2.6. Evaluation of Scratching Behavior

Scratching behavior was evaluated in the context of house dust mite sensitization tests, performed as previously reported, 42 days after the initiation of treatments. AD-indicative behavioral changes, such as nose, ear, or dorsal skin scratching for periods longer than 10 min, were measured and recorded.

### 2.7. Evaluation of Moisture Retention

The amount of moisture retention in the mouse dorsal skin was measured 42 days after the initiation of treatments, at 22 ± 2°C with 50%–55% humidity, using a skin evaporative water recorder (Tewameter TM 300, Courage+Khazaka Electronic, Koln, Germany) and a corneometer (Courage+Khazaka Electronic, Germany). Values were recorded only after signal stabilization, which occurred approximately 10 s after the probe was placed on the skin.

### 2.8. Histological Analysis, Measurement of the Skin Thickness, and Infiltrating Mast Cells

The dorsal skin and one ear per mouse were fixed in 10% (*v*/*v*) neutral buffered formalin for 24 h at 4°C. Tissue samples were embedded in paraffin and sectioned (4 *μ*m thickness). To evaluate tissue architecture, sections were stained with a hematoxylin and eosin (H&E) solution (Sigma-Aldrich, St. Louis, MO, USA) and mounted under coverslips using a Dako mounting medium (DakoCytomation, Glostrup, Denmark). Epidermal and ear thicknesses were quantified by microscopic examination. Briefly, 10 randomly selected areas were observed in H&E-stained preparations (from 3 sections per each animal), under the microscope (Olympus Microscope System CKX53; Olympus, Tokyo, Japan). Representative images were acquired, and thickness measurements were performed using the ImageJ 1.50i software (National Institutes of Health, Bethesda, MD, USA). To measure the degree of mast cell infiltration, sections were stained with toluidine blue (TB), and the number of mast cells was quantified in four fields of view, under the microscope (Olympus Microscope System CKX53; Olympus, Tokyo, Japan). All measurements/quantifications were performed in a blinded manner.

### 2.9. Measurement of Serum IgE, Histamine, and TSLP Levels

The serum levels of IgE (BioLegend, San Diego, CA, USA), histamine (Oxford Biomedical Research, Rochester Hills, MI), and TSLP (R&D Systems Inc., Minneapolis, MN, USA) were measured using commercial enzyme-linked immunosorbent assay (ELISA) kits, in accordance with the manufacturer's instructions.

### 2.10. TSLP Quantification by Immunofluorescence

Dorsal and ear skin sections were washed with phosphate-buffered saline (PBS) prior to immunostaining and then blocked with 0.5% bovine serum albumin for 30 min to prevent nonspecific binding of the antibodies. The sections were then incubated overnight with a primary anti-TSLP antibody (1 : 1000 dilution; Abcam, Cambridge, MA, USA) in PBS containing 0.3% Triton X-100 and normal goat serum and subsequently with a secondary Cy3 conjugate Alexa 594 antibody (1 : 300 dilution; Vector Laboratories, CA, USA) for 60 min. The sections were finally washed in PBS and mounted using a Vectashield mounting medium containing DAPI (Vector Laboratories). The stained sections were observed under the microscope (Olympus Microscope System CKX53). Quantification of the effects was performed by measuring the fluorescence density of TSLP-positive cells at ×40 magnification using an image analyzer (Molecular Devices Inc., CA, USA); data are presented as the percent of the NC group values.

### 2.11. RNA Extraction and Real-Time Reverse Transcription Polymerase Chain Reaction

Dorsal and ear skin tissues were homogenized with a TRIzol reagent (Invitrogen, Carlsbad, CA, USA). Afterwards, 0.2 mL of chloroform was added to each sample, and the tubes were shaken vigorously by hand for 15 s and then incubated at room temperature for 3 min. Mixtures were then centrifuged at 14,000 rpm for 15 min at 4°C, after which the upper aqueous phases (400 *μ*L) were transferred to fresh tubes containing 0.5 mL of 2-propanol. After an incubation step of 10 min at 4°C, the mixtures were centrifuged at 14,000 rpm for 10 min at 4°C, and the supernatants were rejected. The pellets were washed with 1 mL of 75% ethanol and centrifuged again at 10,000 rpm for 5 min at 4°C. The resulting RNA pellets were finally dried and dissolved in diethyl pyrocarbonate-distilled water. Equal amounts of RNA (200 ng) were reversely transcribed into cDNAs using an iScript cDNA synthesis kit (Bio-Rad Laboratories Inc.), according to the manufacturer's protocol. Real-time quantitative polymerase chain reaction (RT-qPCR) analysis was performed according to previously published methods [17, 20]. Primer sequences used in this study were as follows: GAPDH (forward 5′-tgtgaacggatttggccgta-3′, reverse-5′-actgtgccgttgaatttgcc-3′), IL-1*β* (forward 5′-gcagacagctcaatctctaggac-3′, reverse 5′-gctgcctaatgtccccttga-3′), IL-4 (forward 5′-ccatatccacggatgcgaca-3′, reverse 5′-aagcccgaaagagtctctgc-3′), IL-5 (forward 5′-cgtgggggtactgtggaaat-3′, reverse 5′-aatccaggaactgcctcgtc-3′), IL-6 (forward 5′-ccccaatttccaatgctctcc-3′, reverse 5′-cgcactaggtttgccgagta-3′), IL-8 (forward 5′-tgccgtgcaataaccttcat-3′, reverse 5′-aatagagggcatgccagagc-3′), IL-13 (forward 5′-tgccatctacaggacccaga-3′, reverse 5′-ctcattagaaggggccgtgg-3′), TNF-*α* (forward 5′-cccacgtcgtagcaaaccac-3′, reverse 5′-gcagccttgtcccttgaaga-3′), and IFN-*γ* (forward 5′-cggcacagtcattgaaagcc-3′, reverse 5′-tgcatcctttttcgccttgc-3′).

### 2.12. Cell Culture

The human keratinocyte HaCaT cell line was obtained from CLS Cell Lines Service GmbH (Eppelheim, Baden-Württemberg, Germany). HaCaT keratinocytes were maintained and cultured in Dulbecco's modified Eagle's medium (Gibco Inc., Grand Island, NY, USA), supplemented with 10% fetal bovine serum and penicillin (100 U/mL)/streptomycin (100 *μ*g/mL) (all from Gibco Inc., Waltham, MA, USA) in a 5% CO_2_ incubator at 37°C.

### 2.13. Three-Dimensional Human Skin Equivalent System

The three-dimensional human skin equivalent (3HSE) was established according to previously published methods [21, 22]. Using Neoderm®-ED purchased from Tego Science (Seoul, South Korea), human dermal fibroblasts (HDF) were cultured in a collagen matrix for 1 day. Primary keratinocytes were then seeded on top of the collagen matrix and cocultured for 4 days. Next, the primary keratinocytes and HDF blocks were lifted and exposed to air. The 3HSE was treated with CP (31.3 or 62.5 *μ*g/mL) for 1 h. Then, they were stimulated with TNF-*α*/IFN-*γ* (10 ng/mL) or monosodium urate crystal (MSU, 0.1 or 0.5 mg/mL) for an additional 23 h. The skin equivalent was incubated at 37°C under 5% CO_2_, and the cell culture system was then established according to previously published methods [21, 22].

### 2.14. Skin Histological Analysis of 3HSE

Skin sections from the 3HSE were prepared for hematoxylin and eosin staining. Sections (5 *μ*m thick) of 10% neutral formalin solution-fixed paraffin-embedded tissues were cut on saline-coated glass slides and then deparaffinized 3 times with xylene and dehydrated through a graded alcohol bath. The deparaffinized sections were stained with hematoxylin for 5 min. The slides were then washed and stained with EosinY. Finally, they were dehydrated and washed. Representative images were taken using a fluorescence microscope (Olympus Microscope System CKX53).

### 2.15. Measurement of Reactive Oxygen Species

Treated and control HaCaT cells were incubated with 20 *μ*M 2′,7′-dichlorofluorescein diacetate (DCFDA; Sigma-Aldrich, St. Louis, MO, USA) for 30 min at 37°C. DCF fluorescence was determined by SpectraMax i3 Multi-Mode Detection Platform (Molecular Devices, LLC., CA, USA).

### 2.16. Western Blot Analysis

Dorsal and ear skin samples and HaCaT keratinocytes were lysed in RIPA lysis and extraction buffer (Thermo Fisher Scientific, Rockford, IL, USA) containing a protease inhibitor cocktail (cOmplete™; Roche, Mannheim, Germany). Protein concentrations were determined in tissue extracts using the Bradford method (Bio-Rad Laboratories Inc., Hercules, CA, USA) and bovine serum albumin (BSA) as the standard. Equal amounts of protein extracts (30 *μ*g) were resolved by 12% or 4%–20% sodium dodecyl sulfate-polyacrylamide gel electrophoresis and transferred to a polyvinylidene fluoride membrane (Bio-Rad Laboratories Inc., Hercules, CA, USA), using the transfer system YYY. Membranes were blocked with 3% BSA in Tris-buffered saline containing Tween 20 (TBST), followed by an overnight incubation step at 4°C with the appropriate primary antibodies. Antibodies used included anti-Aquaporin 3 (Abcam, Cambridge, UK), anti-NLRP3, anti-ASC, anti-cleaved caspase-1, anti-IL-1*β* (all from Cell Signaling Technology, Danvers, MA, USA), and anti-*β*-actin (Santa Cruz Biotechnology, Dallas, TX, USA). The membranes were then washed three times with TBST and incubated with a horseradish peroxidase- (HRP-) conjugated secondary antibody (Jackson ImmunoResearch, West Grove, PA, USA) for 1 h at room temperature. The membranes were again washed three times with TBST, and immunoreactivity was then visualized using an enhanced chemiluminescence kit (Thermo Fisher Scientific, Waltham, MA, USA). Images were captured using the ChemiDoc XRS+ system (Bio-Rad Laboratories Inc., Hercules, CA, USA). Relative expression levels were obtained based on the expression of *β*-actin, using the ChemiDoc Band Analysis system (Bio-Rad Laboratories Inc., Hercules, CA, USA).

### 2.17. Statistical Analysis

Generally, data are presented as group means ± standard error of the mean (SEM). Statistical analysis was performed using the GraphPad Prism 7.0 software (GraphPad Software, San Diego, CA, USA). Statistical comparisons were performed using the one-way analysis of variance (ANOVA) test, with Dunnett's post hoc analysis. A value of *P* < 0.05 was considered statistically significant.

## 3. Results

### 3.1. CP Treatments Ease the House Dust Mite-Induced AD-Like Symptoms in NC/Nga Mice

To understand the therapeutic potential of CP in the context of AD, we administered different CP concentrations to a mouse model of house dust mite-induced AD and evaluated disease development, progression, and severity, by measuring dermatitis scores, skin moisture retention, and animal behavioral changes. As expected, the untreated control group presented a significantly increased dermatitis score than the negative control group. Interestingly, animals treated with CP 1 and 3% preparations showed less severe dermatitis scores, significantly lower than the ones obtained for the untreated control group (Figure 1(b)). These results were in line with the animal behavioral changes recorded. Scratching frequency was significantly higher, comparing the untreated control group with the negative control group (Figure 1(c)). Additionally, the groups of animals treated with CP 1 and 3% preparations showed a reduction in scratching times (Figure 1(c)). Also, moisture retention measurements showed an intermediate phenotype, particularly in animals treated with CP 3% preparation. The corneometer score was significantly higher in this group, compared to untreated control animals (Figure 1(d)). Importantly, the positive control Dex-0.1% group was the one that showed less severe AD-like symptoms. Overall, these results suggest that CP treatments have an alleviatory effect in AD clinical symptoms *in vivo*.

### 3.2. CP Treatments Lead to Less Severe Pathologic Outcomes in a House Dust Mite-Induced AD Mouse Model

**Figure 2:**
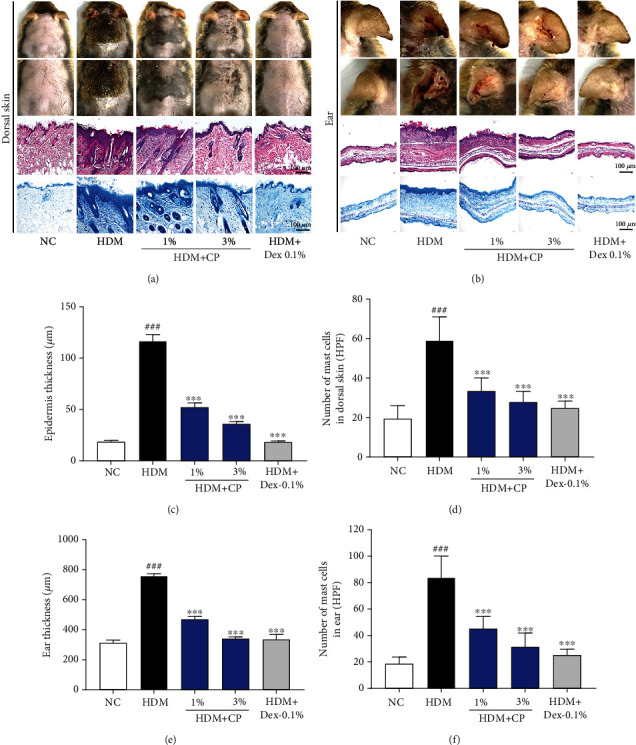
CP treatments decrease pathology in a house dust mite-induced AD mouse model. Histological features of mouse (a) dorsal and (b) ear skin preparations. Tissues were excised, fixed in 10% formaldehyde, embedded in paraffin, and sectioned. Sections were stained with H&E (scale bar = 100 *μ*m) or with toluidine blue to identify mast cells (scale bar = 100 *μ*m). (c) Epidermal and (e) ear thicknesses in H&E-stained sections were measured under a microscope. Mast cells were counted in toluidine blue-stained sections of (d) dorsal and (f) ear skin tissues, under a microscope. Dexamethasone (Dex) was used as a positive control. All data are represented per group, as means ± SEM of three independent experiments. Statistical differences were evaluated using the one-way ANOVA test, with Dunnett's post hoc analysis, and are represented as follows: ^###^*P* < 0.001 compared with the negative control group (NC); ^∗∗∗^*P* < 0.001 compared with the untreated control group (HDM).

To understand if the milder symptomatology recorded for CP-treated groups was a consequence of less severe pathologies, we performed histological analysis of dorsal and ear skin sections stained with H&E and TB. The H&E stainings suggested that the untreated control group displayed skin hyperkeratosis (Figures 2(a) and 2(b)). Importantly, the degree of histologic severity aligned with the abovementioned different severities. The epidermal and ear thickness values were higher for the untreated control animals, compared with the negative control group (Figures 2(c) and 2(e)). Once more, animals treated with CP 1 and 3% preparations showed an intermediate phenotype, less severe than the one observed for the untreated control group: substantially reduced epidermal and ear thicknesses (Figures 2(c) and 2(e)). With respect to mast cell infiltration, a known marker of inflammation, results followed the same pattern. The untreated control animals showed the highest frequencies of infiltrating cells, followed by the animals treated with CP 1 and 3% preparations that showed an intermediate phenotype, followed by the negative control group (Figures 2(d) and 2(f)). Once again, treatments with Dex-0.1% preparation suppressed the increase of epidermis and ear thickness, as well as the infiltration of mast cells. These results suggest that the topical administration of CP may prevent the development of severe pathological states of AD *in vivo*.

### 3.3. CP Treatments Promote the Decrease of IgE Serum Levels, Histamine Release, and Aquaporin Expression 3 in a House Dust Mite-Induced AD Mouse Model

**Figure 3:**
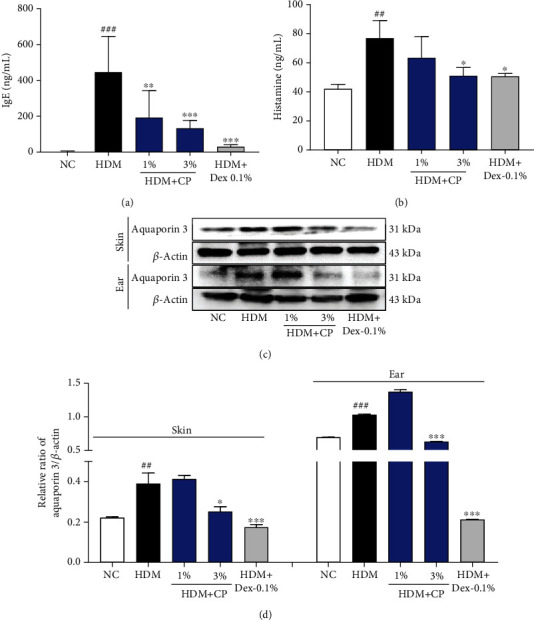
CP treatment effects on IgE and histamine systemic levels and local aquaporin 3 expression in a house dust mite-induced AD mouse model. (a) IgE and (b) histamine serum levels were measured by ELISA. (c) The expression of aquaporin 3 was detected by Western blot, in dorsal and ear skin samples. *β*-Actin protein was used as an internal control. (d) Relative expression of aquaporin 3 was quantified. Dexamethasone (Dex) was used as a positive control. All data are represented per group, as means ± SEM of three independent experiments. Statistical differences were evaluated using the one-way ANOVA test, with Dunnett's post hoc analysis, and are represented as follows: ^##^*P* < 0.01 and ^###^*P* < 0.001 compared with the negative control group (NC); ^∗^*P* < 0.05, ^∗∗^*P* < 0.01, and ^∗∗∗^*P* < 0.001 compared with the untreated control group (HDM).

To investigate the effect of CP treatments on the main AD clinical features, we measured the levels of IgE and histamine in serum. The untreated control group showed significantly increased serum IgE and histamine levels, compared to the negative control group (Figure 3(a)). Remarkably, animals treated with CP 1% and 3% preparations showed significantly decreased serum IgE levels, compared with the untreated control group (Figure 3(a)). Additionally, only the group of animals treated with the CP 3% preparation showed lower levels of serum histamine, compared with the untreated control group (Figure 3(b)). To confirm the involvement of aquaporin 3 in the epidermal hyperplasia and barrier disruption (known AD characteristics) observed, in different extents in the abovementioned histological preparations, we quantified aquaporin 3 expression levels in dorsal and ear skin samples. As shown in Figures 3(c) and 3(d), the levels of aquaporin 3 expression were significantly decreased in animals that received the CP 3% treatments, compared to those determined for the untreated control group. Once more, the positive control group (Dex-0.1%-treated animals) showed the expected phenotype, promoting decreased IgE and histamine systemic levels and reduced local aquaporin 3 expression. Overall, these results suggest that CP has an anti-AD effect, preventing the release of AD-related inflammatory mediators and the development of epidermal hyperplasia states *in vivo*.

### 3.4. CP Treatments Inhibit the Expression of TSLP in a House Dust Mite-Induced AD Mouse Model

**Figure 4:**
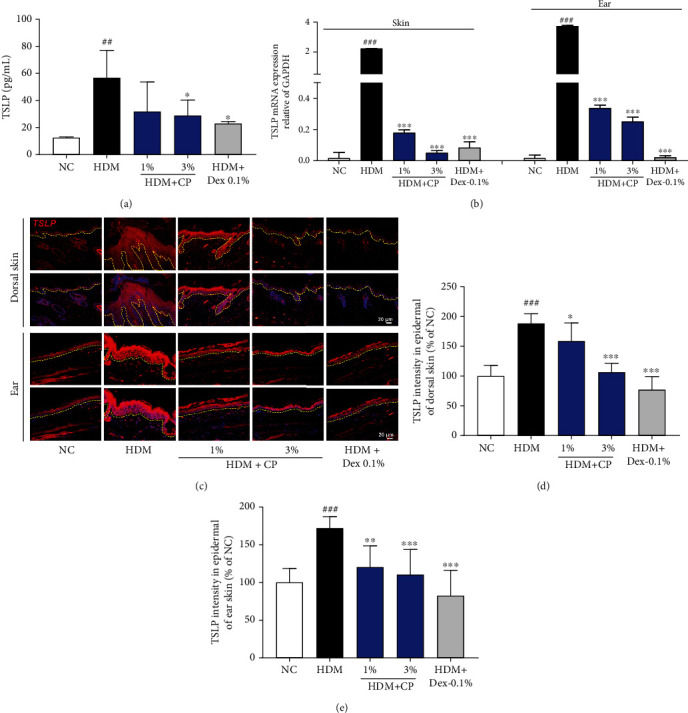
CP treatment effect on TSLP secretion and mRNA expression in a house dust mite-induced AD mouse model. (a) TSLP serum levels were measured by ELISA. (b) The relative expression of TSLP was analyzed by real-time PCR, in dorsal and ear skin samples, normalized to GAPDH. (c) TSLP levels were also evaluated locally, by immunofluorescence analysis, for evaluation in dorsal and ear skin samples.Scale bar = 20 *μ*m. Quantitative analysis for (d) dorsal and (e) ear skin TSLP levels is also represented. Dexamethasone (Dex) was used as a positive control. All data are represented per group, as means ± SEM of three independent experiments. Statistical differences were evaluated using the one-way ANOVA test, with Dunnett's post hoc analysis, and are represented as follows: ^##^*P* < 0.01 and ^###^*P* < 0.001 compared with the negative control group (NC); ^∗^*P* < 0.05, ^∗∗^*P* < 0.01, and ^∗∗∗^*P* < 0.001 compared with the untreated control group (HDM).

To investigate the effect of CP treatments on AD-related immune responses, we measured the production and expression of TSLP, an essential mediator of T cell maturation, in the different groups of mice induced for AD development. As can be observed in Figure 4(a), in serum, TSLP levels were significantly increased, comparing the untreated control group with the negative control group. Importantly, CP 1 and 3% treatments affected systemic TSLP levels: lower serum levels were detected in a tendential or significant fashion, respectively, in comparison with the untreated control group. These differences were also observed locally. The TSLP mRNA expression levels, measured in skin samples by RT-qPCR, were also significantly increased in untreated controls, compared with the negative control animals. Furthermore, CP 1% and 3% groups indicated a decrease greater than that in the AD group (Figure 4(b)). These phenotypes were confirmed by TSLP skin immunofluorescence analysis (Figures 4(c)–4(e)). The Dex-0.1% group also obtained significant results in TSLP mRNA expression and immunofluorescence analysis. Together, these results demonstrate that CP treatments potentially impact the development of AD-related immune responses via the regulation of TSLP expression and secretion.

### 3.5. CP Treatments Modulate the Activation of the NLRP3 Inflammasome in a House Dust Mite-Induced AD Mouse Model

**Figure 5:**
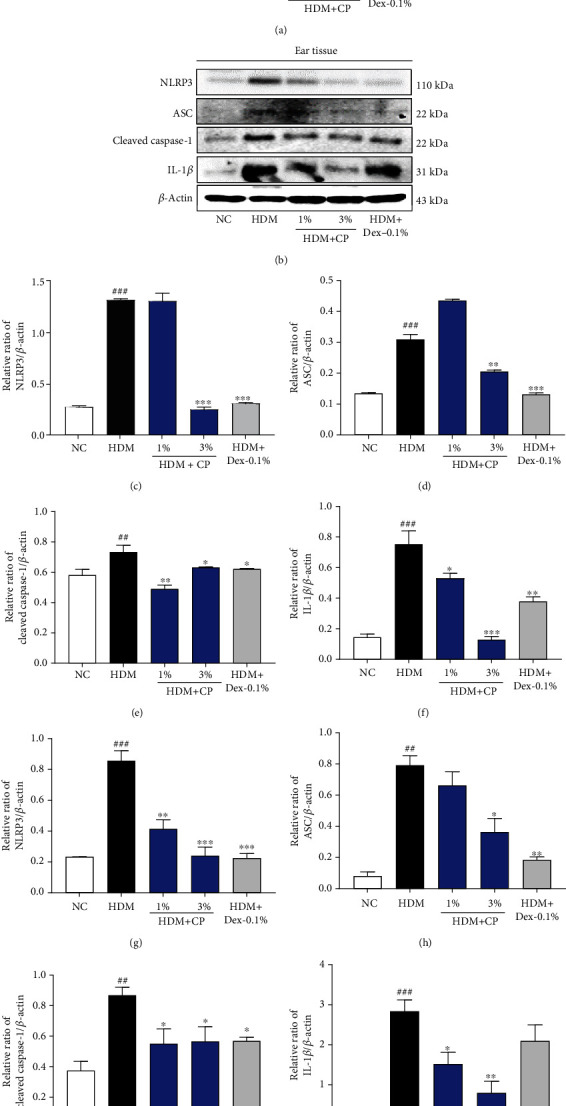
CP treatment affects NLRP3 inflammasome activation in a house dust mite-induced AD mouse model. (a) NLRP3 inflammasome components were analyzed in dorsal skin samples by Western blot. Bar graphs represent the relative expression of (c) NLRP3, (d) ASC, (e) cleaved caspase-1, and (f) IL-1*β*. (b) NLRP3 inflammasome components were also analyzed in ear skin samples. Bar graphs represent the relative expression of (g) NLRP3, (h) ASC, (i) cleaved caspase-1, and (j) IL-1*β*. Relative expression levels were obtained by normalization against *β*-actin. Dexamethasone (Dex) was used as a positive control. All data are represented per group, as means ± SEM of three independent experiments. Statistical differences were evaluated using the one-way ANOVA test, with Dunnett's post hoc analysis, and are represented as follows: ^##^*P* < 0.01 and ^###^*P* < 0.001 compared with the negative control group (NC); ^∗^*P* < 0.05, ^∗∗^*P* < 0.01, and ^∗∗∗^*P* < 0.001 compared with the untreated control group (HDM).

The notion that CP treatments potentially modulate AD-related immune responses, together with the fact that inflammasomes and AD are strictly related, led us to focus on NLRP3 inflammasome activation and quantify the relevant mediators/byproducts. As shown in Figures 5(a)–5(e), the protein levels of ASC, cleaved caspase-1, and IL-1*β* were significantly increased in dorsal skin tissues from untreated control animals, compared to the negative control group. Importantly, animals that received CP 1 and 3% treatments showed lower expression levels of ASC, cleaved caspase-1, and IL-1*β* than untreated control animals. These results were completely reproduced by the ones obtained in the analysis of ear skin samples (Figures 5(f)–5(j)). Altogether, these data suggest that CP treatments inhibit NLRP3 inflammasome activation, modulating AD-related immune responses.

### 3.6. CP Treatments Impact Inflammatory Cytokine Gene Expression in a House Dust Mite-Induced AD Mouse Model

**Figure 6:**
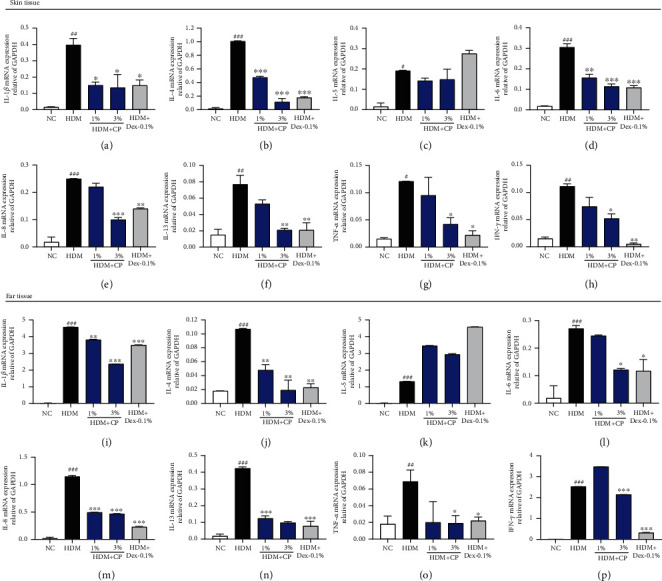
CP treatment effect on the expression of inflammatory cytokines in a house dust mite-induced AD mouse model. The relative expression of Th1/Th2 inflammatory cytokines was analyzed by real-time PCR in dorsal and ear skin samples. Values were normalized to GAPDH expression levels. Normalized results for (a) IL-1*β*, (b) IL-4, (c) IL-5, (d) IL-6, (e) IL-8, (f) IL-13, (g), TNF-*α*, and (h) IFN-*γ* expression in dorsal skin samples are shown. Normalized results for (i) IL-1*β*, (j) IL-4, (k) IL-5, (l) IL-6, (m) IL-8, (n) IL-13, (o) TNF-*α*, and (p) IFN-*γ* expression in ear skin samples are also represented. Dexamethasone (Dex) was used as a positive control. All data are represented per group, as means ± SEM of three independent experiments. Statistical differences were evaluated using the one-way ANOVA test, with Dunnett's post hoc analysis, and are represented as follows: ^#^*P* < 0.05, ^##^*P* < 0.01, and ^###^*P* < 0.001 compared with the negative control group (NC); ^∗^*P* < 0.05, ^∗∗^*P* < 0.01, and ^∗∗∗^*P* < 0.001 compared with the untreated control group (HDM).

The reported differences observed with respect to TSLP levels and inflammasome activation dictated a broader investigation of AD-related inflammatory/immune responses. Consequently, we evaluated cytokine gene expression patterns in dorsal and ear skin samples by RT-qPCR. First, the results obtained for IL-1*β* expression in dorsal (Figure 6(a)) and ear (Figure 6(i)) skin samples support the abovementioned results and the hypothesis that CP treatments inhibit inflammasome activation. However, this was not the only proinflammatory cytokine differentially expressed in the context of CP treatments. The gene expression levels of TNF-*α* and IFN-*γ*, like IL-1*β*, were significantly higher in untreated control animal skin samples, compared to the negative control group (Figures 6(g) and 6(h) and Figures 6(o) and 6(p), for dorsal and ear skin, respectively). Additionally, and once more, CP 1% and 3% treatments downregulated the expression of TNF-*α* and IFN-*γ* (Figures 6(g) and 6(h) and Figures 6(o) and 6(p), for dorsal and ear skin, respectively, compared to the untreated control group). With respect to Th2 cytokines, different expression patterns were also detected. IL-4, IL-5, IL-6, IL-8, and IL-13 expression levels were significantly increased in untreated control animal skin samples, compared to the negative control group. Curiously, comparing samples from CP-treated groups with the ones from untreated control animals, all but IL-5 gene expression levels were lower (Figures 6(b)–6(f) and Figures 6(j)–6(n), for dorsal and ear skin, respectively). These results overall point to the hypothesis that CP treatments control the magnitude of AD-related Th1/Th2 local immune responses.

### 3.7. CP Inhibits NLRP3 Inflammasome Activation *In Vitro*

**Figure 7:**
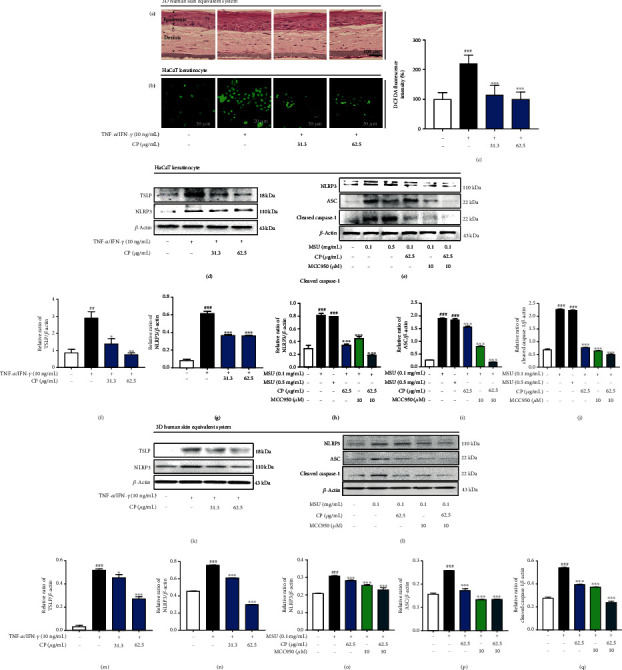
CP treatments modulate ROS production and NLRP3 inflammasome activation *in vitro*. Two different NLRP3 inflammasome activators, TNF-*α*/IFN-*γ* or MSU, were used in HaCaT keratinocyte and 3HSE. (a) Hematoxylin and eosin staining was used to identify structures in cells and tissues, in 3HSE stimulated with TNF-*α*/IFN-*γ*, in the presence or absence of CP (31.3 or 62.5 *μ*g/mL). (b, c) ROS levels were measured by the DCFDA assay, in HaCaT cells stimulated with TNF-*α*/IFN-*γ*, in the presence or absence of CP (31.3 or 62.5 *μ*g/mL). (d) TSLP and NLRP3 expression levels were measured by Western blot in HaCaT cells. Normalized values of (f) TSLP and (g) NLRP3 expression are represented. (e) NLRP3, ASC, and cleaved caspase-1 expression levels were also measured in HaCaT cells pretreated (or not) with CP (62.5 *μ*g/mL) or MCC950 (10 *μ*M) and stimulated with monosodium urate crystal (MSU). Western blot was performed to determine the effect of CP on the expression of NLRP3 inflammasome activation. Results were normalized and are represented for (h) NLRP3, (i) ASC, and (j) cleaved caspase-1 for (e). (k) TSLP and NLRP3 expression levels were measured by Western blot in 3HSE. Normalized values of (m) TSLP and (n) NLRP3 expression are represented. (l) NLRP3, ASC, and cleaved caspase-1 expression levels were also measured in 3HSE pretreated (or not) with CP (62.5 *μ*g/mL) or MCC950 (10 *μ*M) and stimulated with monosodium urate crystal (MSU). Western blot was performed to determine the effect of CP on the expression of NLRP3 inflammasome activation. Results were normalized and are represented for (o) NLRP3, (p) ASC, and (q) cleaved caspase-1 for (l). MCC950 is an inhibitor of NLRP3. All data are represented per group, as means ± SEM of three independent experiments. Statistical differences were evaluated using the one-way ANOVA test, with Dunnett's post hoc analysis, and are represented as follows: ^##^*P* < 0.01 and ^###^*P* < 0.001 compared with nontreated cells; ^∗^*P* < 0.05 and ^∗∗∗^*P* < 0.001 compared with TNF-*α*/IFN-*γ*- or MSU-treated cells.

To ultimately prove the CP inhibitory effect on NLRP3 inflammasome responses and derive a definitive mechanistic insight into our study, we evaluated *in vitro* the effect of CP treatments on two different systems of NLRP3 inflammasome activation in HaCaT keratinocytes and 3HSE. CP was treated *in vitro* at concentrations ranging from 31.3 to 1000 *μ*g/mL. CP did not affect cell viability at doses ranging from 31.3 to 62.5 *μ*g/mL. A nontoxic concentration (31.3 or 62.5 *μ*g/mL) was used in subsequent experiments [23]. First, hematoxylin and eosin staining was performed in 3HSE; as shown in Figure 7(a), CP did not induce human skin keratinocyte and fibroblast cell toxicity. For *in vitro* experiments, CP was used at a nontoxic concentration. We measured intracellular reactive oxygen species (ROS) generation and TSLP protein expression levels. When we used TNF-*α*/IFN-*γ* as induction conditions, ROS levels were significantly higher in induced versus noninduced cells compared to untreated cells in HaCaT keratinocyte (Figure 7(b)). Interestingly, when the inflammasome was induced in the presence of CP, ROS-generated levels decreased (Figure 7(c)). These results may suggest that CP can effectively scavenge ROS. Furthermore, in line with the results obtained *in vivo*, CP-treated cells showed significantly reduced TSLP protein expression levels, compared with TNF-*α*/IFN-*γ*-induced nontreated cells. Furthermore, TNF-*α*/IFN-*γ*-induced NLRP3 inflammasome activation was also significantly decreased in CP-treated cells (Figures 7(d), 7(f), and 7(g)). To further confirm these results, we used an alternative NLRP3 activator, MSU, in two different doses (0.1 or 0.5 mg/mL). Once again, inflammasome induction was confirmed, comparing with the noninduced condition, to a similar extent for the 2 MSU concentrations. Additionally, the CP treatment-induced NLRP3 inflammasome inhibition was again proved, by the significant decrease in NLRP3, ASC, and cleaved caspase-1 protein levels, detected in a dose-dependent manner. We evaluated the action of CP when used in combination with MCC950, a selective inhibitor of NLRP3. In this context, we observed an additive NLRP3 inflammasome inhibitory effect suggesting that indeed CP has inflammasome inhibitory properties (Figures 7(e), 7(h)–7(j)). Finally, in 3HSE, inhibition of TSLP and NLRP3 inflammasome activation by CP was confirmed similar to the results of HaCaT cells (Figures 7(k)–7(q)). These *in vitro* results ultimately suggest that the milder AD phenotypes observed *in vivo* in the groups of animals treated with CP preparations are a result of direct inflammasome inhibition.

## 4. Discussion

Crude drugs have been explored for decades, as safer alternatives to synthetic pharmaceutics, and used for thousands of years to treat various diseases in Asian countries [24]. Many studies have highlighted the importance of the delivery routes used in treatments with crude drugs to target relevant tissues and achieve the expected therapeutic effects in animal models of disease [25–27]. Two routes of administration are widely used in AD models: topical and oral. Although by the topical administration route the control of the therapeutic dosage is always a challenge, the close contact of crude drugs with the target tissues ensures a relatively fast action [28–30]. Therefore, topical administration of effective crude drugs, with no side effects, should be the preferred route used for the treatment of AD. In the present study, we used this route to test the potential of CP, a crude drug known as a cast-off skin of cicada, as a therapy for AD.

The primary etiology of AD is allergic sensitization, with IgE hypersecretion, inflammatory infiltration, and Th1/Th2 response imbalance [31]. The clinical symptoms of AD include dryness, itching, erythema, and edema [31]. The NC/Nga mouse model is a well-established spontaneous model of human AD [32, 33]. However, the development of spontaneous AD-like lesions in this model is of late onset and has a relatively low incidence of less than 50% [34]. Thus, we used a house dust mite, a common allergen, to warrant and accelerate AD development in NC/Nga mice. Importantly, it is known that the repeated mouse skin exposure to house dust mites leads to hyperkeratosis, thickening of the epidermis, and infiltration of lymphocytes and mast cells, all characteristics similar to human AD presentations. In this study, we found that the topical application of CP attenuated house dust mite-induced AD-like skin lesion development in NC/Nga mice. CP treatment improved hyperkeratosis states, skin lesion severity, dermatitis scores, and animal scratching behavior. Overall, these findings suggest that topical administration of CP may be useful for the treatment of AD.

AD is characterized by skin barrier defects. Previous reports showed that transepidermal water loss in skin lesions was significantly increased in patients who had a disturbed skin barrier [35]. Aquaporins are a family of transmembrane channels that transport water and, in some cases, small solutes such as glycerol [36]. Aquaporin 3 is expressed by epidermal keratinocytes, known to upregulate its expression in response to stress or damage (e.g., induced by retinoic acid) as well as in the context of diseases such as atopic eczema and skin carcinomas [37]. On the other hand, in some dermatologic conditions, such as psoriasis and lesions, aquaporin 3 levels are often lower than in healthy skin [38]. In this study, we observed that CP treatments attenuated the loss of the skin barrier in a mouse model of AD. We hypothesize that the stratum corneum hydration levels were maintained by the downregulation of aquaporin 3 protein expression. Overall our results suggest that CP treatment promotes skin barrier integrity/function and moisture retention and consequently preserves normal keratinocyte functions, preventing AD pathological consequences.

Increased serum IgE levels are frequently detected secondarily to acute and chronic atopic skin manifestations [1]. Additionally, the repeated application of house dust mites to the NC/Nga mouse skin was showed to induce IgE secretion, as we also describe here [1, 33]. IgE overproduction activates IgE-mediated expression of Th1 and Th2 cytokines, and AD typical immune responses are associated with inflammatory infiltrates composed of lymphocytes, mast cells, dendritic cells, and macrophages, which secrete cytokines such as IL-4, IL-13, and IL-31 [39, 40]. While the Th2 cytokine IL-4 is important for acute atopic eczema development, the Th1 and Th17 cytokines IFN-*γ*, IL-17, and IL-22 predominate in chronic eczema states [3, 39]. Under normal conditions, Th1 and Th2 responses are mutually regulated [40]. Here, we demonstrated that CP treatments inhibit mast cell infiltration and its allergic effects. Moreover, serum IgE and histamine levels were reduced by CP administration, as were local mRNA levels of several Th1/Th2 inflammatory cytokines, including IL-4, IL-6, IL-8, IL-13, TNF-*α*, and IFN-*γ*, measured in AD-like lesions. These results attest to the potential of CP treatments as immune response attenuators via the regulation of cell infiltration events and cytokine secretion.

TSLP is one of several mediators that can trigger Th2 differentiation and was recently showed to play a critical role in the progression of AD, precisely through the induction of Th2 immune responses [41]. Indeed, high levels of TSLP expression have been observed in epidermal keratinocytes of AD skin lesions [42]. Therefore, suppressing TSLP effects and/or downregulating TSLP levels may represent novel therapeutic approaches for the treatment of AD, by restoring Th1/Th2 balance. Here, we found that CP treatments reduced the levels of TSLP both *in vivo*, in a house dust mite-induced AD mouse model, and *in vitro*, in TNF-*α*/IFN-*γ*-treated HaCaT keratinocytes and 3HSE. The effect of CP treatments on TSLP levels supports the hypothesis that this crude drug has immunoregulatory properties.

**Figure 8:**
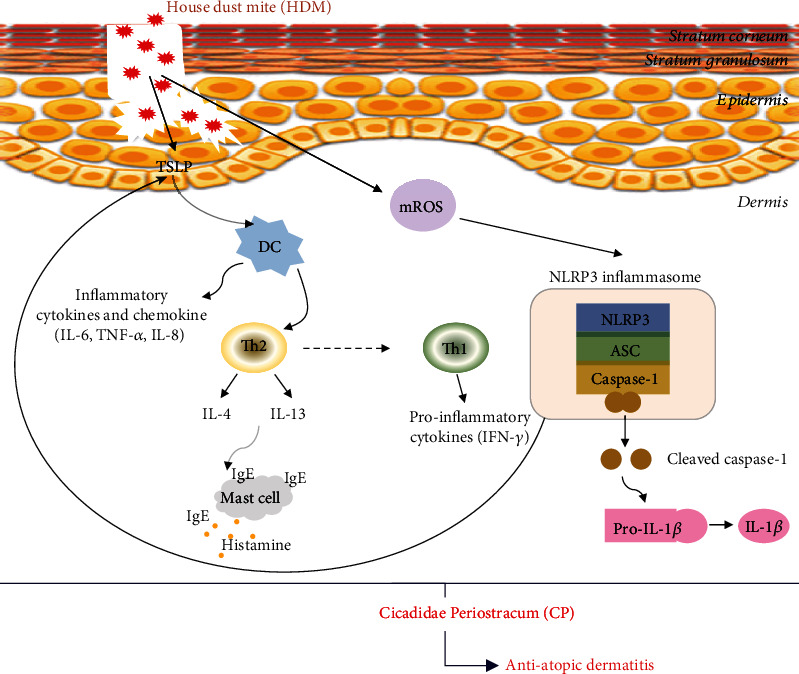
Schematic model of CP treatment effects on a house dust mite-induced AD mouse model. Upon house dust mite exposure, epithelial barrier disturbances, functional defects, and inflammatory cell infiltrates were observed. The barrier-disrupted epidermis abundantly releases thymic stromal lymphopoietin (TSLP), which triggers the magnification of Th1/Th2 immune responses. CP treatments reduced AD severity, relieved AD-like symptoms, and attenuated inflammatory cell infiltration and Th1/Th2 cytokine expression. CP treatments potentially prevent AD through the inhibition of NLRP3 inflammasome activation via the impairment of ROS production/function.

The inflammasome is a multiprotein complex, consisting of a sensor protein, such as NLRs, an adaptor protein (ASC), and a cleaving protein (caspase-1) [9]. The NLRP3 inflammasome is triggered by various physical and chemical stimuli and drives IL-1*β* and IL-18 activation and release and the initiation/amplification of a series of inflammatory responses [9, 10]. Importantly, the NLRP3 inflammasome plays a crucial role in AD development [43]. Endogenous or exogenous pathogens are recognized inflammasome-triggering factors, directly or via the generation of ROS [44]. ROS may lead to programmed cell death, by increasing the cytosolic concentration of proapoptotic factors, but are also crucial NLRP3 inflammasome activators [44, 45]. Here, we investigated the effect of CP on NLRP3 inflammasome activation. We found that CP treatments resulted in lower ROS production/availability, impacting the TNF-*α*/IFN-*γ*-induced oxidative stress and cellular redox status of HaCaT keratinocytes. Furthermore, we demonstrated that NLRP3 inflammasome activation was significantly downregulated after CP treatments *in vitro* and *in vivo*. Additionally, NLRP3 inflammasome activation, induced by MSU treatment, was affected by CP treatment in HaCaT keratinocytes and 3HSE. 3HSE is used to characterize the mode of action of novel agents and their efficacy in the skin; it is regarded as a valid alternative for animal testing with numerous applications. Taken together, these findings provide evidence that CP can effectively ameliorate AD-like symptoms via the modulation of TSLP expression and NLRP3 inflammasome activation, impacting Th1/Th2 immune response (Figure 8). Additionally, our results strongly suggest that NLRP3 inflammasome activation is a trigger of dermatitis.

In conclusion, this study demonstrates that CP has anti-AD therapeutic potential. Topical administration of CP has been successfully used to treat a variety of peripheral tissue wounds effectively and with excellent tolerance. Here, CP topical administration inhibited mast cell hyperplasia, a major process in the induction of atopic dermatitis. Additionally, CP treatments highly ameliorated house dust mite-induced AD-like skin inflammatory responses *in vivo*. These findings suggest that CP treatments reduced the expression levels of TSLP and Th1/Th2 cytokines likely via the NLRP3 inflammasome activation-inhibition, in AD-like skin lesions. However, to fully understand the mechanisms behind the ease in AD symptomatology and the full potential of CP as a therapy for AD, additional preclinical (and even clinical) research studies are necessary.

## Data Availability

The data used to support the findings of this study are available from the corresponding author upon request.
